# Supplementary material for: Modeling glioblastoma heterogeneity as a dynamic network of cell states
Source: Mol Syst Biol. 2021 Sep 16;17(9):e10105. doi: 10.15252/msb.202010105 (PMC8444284; doi:10.15252/msb.202010105)
Supplement: Supplementary file 5 — Source Data for Figure 3 [file MSB-17-e10105-s001.zip › Figure3A_sourcedata/GSEA_3065/nosigdb_state5.GseaPreranked.1622718435914/LARSSON_STATE_6.html]

Details for gene set LARSSON\_STATE\_6[GSEA]

|  || Dataset | state5 |
| Phenotype | NoPhenotypeAvailable |
| Upregulated in class | na\_neg |
| GeneSet | LARSSON\_STATE\_6 |
| Enrichment Score (ES) | -0.7642124 |
| Normalized Enrichment Score (NES) | -2.6547146 |
| Nominal p-value | 0.0 |
| FDR q-value | 0.0 |
| FWER p-Value | 0.0 |
Table: GSEA Results Summary

  

Fig 1: Enrichment plot: LARSSON\_STATE\_6      
 Profile of the Running ES Score & Positions of GeneSet Members on the Rank Ordered List

  

| PROBE | GENE SYMBOL | GENE\_TITLE | RANK IN GENE LIST | RANK METRIC SCORE | RUNNING ES | CORE ENRICHMENT || 1 | NEAT1 |  |  | 17 | 0.830 | 0.0171 | No |
| 2 | CLU |  |  | 162 | 0.474 | 0.0117 | No |
| 3 | DKK3 |  |  | 1050 | 0.204 | -0.0834 | No |
| 4 | SPARCL1 |  |  | 1663 | 0.136 | -0.1492 | No |
| 5 | HNRNPA2B1 |  |  | 1852 | 0.122 | -0.1676 | No |
| 6 | EFTUD2 |  |  | 4927 | -0.030 | -0.5129 | No |
| 7 | CD74 |  |  | 5224 | -0.041 | -0.5453 | No |
| 8 | QKI |  |  | 5715 | -0.062 | -0.5990 | No |
| 9 | IRS2 |  |  | 6033 | -0.076 | -0.6329 | No |
| 10 | FOXO3 |  |  | 6238 | -0.087 | -0.6539 | No |
| 11 | PBX1 |  |  | 6551 | -0.105 | -0.6866 | No |
| 12 | TSC22D4 |  |  | 6599 | -0.108 | -0.6894 | No |
| 13 | HNRNPDL |  |  | 6625 | -0.109 | -0.6897 | No |
| 14 | FAM20C |  |  | 6750 | -0.117 | -0.7010 | No |
| 15 | FYN |  |  | 6823 | -0.123 | -0.7063 | No |
| 16 | SESN3 |  |  | 6935 | -0.132 | -0.7158 | No |
| 17 | ZMAT3 |  |  | 6960 | -0.134 | -0.7154 | No |
| 18 | TXNIP |  |  | 7037 | -0.140 | -0.7208 | No |
| 19 | GABBR2 |  |  | 7041 | -0.141 | -0.7179 | No |
| 20 | C4orf48 |  |  | 7110 | -0.146 | -0.7222 | No |
| 21 | NFIB |  |  | 7111 | -0.146 | -0.7188 | No |
| 22 | GFAP |  |  | 7113 | -0.146 | -0.7156 | No |
| 23 | COL4A3BP |  |  | 7195 | -0.155 | -0.7212 | No |
| 24 | ID2 |  |  | 7333 | -0.171 | -0.7327 | No |
| 25 | HEY1 |  |  | 7370 | -0.177 | -0.7327 | No |
| 26 | PLEKHB1 |  |  | 7506 | -0.191 | -0.7435 | No |
| 27 | OLFM2 |  |  | 7624 | -0.207 | -0.7519 | No |
| 28 | TNFRSF21 |  |  | 7627 | -0.208 | -0.7474 | No |
| 29 | MXD4 |  |  | 7641 | -0.209 | -0.7441 | No |
| 30 | ID1 |  |  | 7821 | -0.238 | -0.7588 | Yes |
| 31 | RND2 |  |  | 7823 | -0.239 | -0.7534 | Yes |
| 32 | MAP1LC3A |  |  | 7856 | -0.245 | -0.7514 | Yes |
| 33 | PNRC1 |  |  | 7867 | -0.247 | -0.7469 | Yes |
| 34 | CEBPD |  |  | 7899 | -0.256 | -0.7445 | Yes |
| 35 | SOX4 |  |  | 7958 | -0.268 | -0.7449 | Yes |
| 36 | CITED1 |  |  | 8014 | -0.278 | -0.7447 | Yes |
| 37 | SEPT7 |  |  | 8018 | -0.279 | -0.7387 | Yes |
| 38 | MAGED1 |  |  | 8045 | -0.284 | -0.7351 | Yes |
| 39 | CTNNA1 |  |  | 8047 | -0.284 | -0.7287 | Yes |
| 40 | PMP2 |  |  | 8058 | -0.288 | -0.7232 | Yes |
| 41 | NLRP1 |  |  | 8106 | -0.298 | -0.7217 | Yes |
| 42 | TTC3 |  |  | 8127 | -0.303 | -0.7170 | Yes |
| 43 | SAT1 |  |  | 8162 | -0.314 | -0.7137 | Yes |
| 44 | FHL1 |  |  | 8198 | -0.326 | -0.7102 | Yes |
| 45 | TUBB2B |  |  | 8224 | -0.332 | -0.7054 | Yes |
| 46 | MARCH2 |  |  | 8250 | -0.339 | -0.7004 | Yes |
| 47 | HOPX |  |  | 8257 | -0.341 | -0.6933 | Yes |
| 48 | BNIP3L |  |  | 8266 | -0.344 | -0.6863 | Yes |
| 49 | MT3 |  |  | 8279 | -0.348 | -0.6797 | Yes |
| 50 | CRYAB |  |  | 8315 | -0.358 | -0.6754 | Yes |
| 51 | SOX9 |  |  | 8327 | -0.361 | -0.6684 | Yes |
| 52 | C1QL1 |  |  | 8355 | -0.371 | -0.6629 | Yes |
| 53 | TUBB2A |  |  | 8364 | -0.376 | -0.6552 | Yes |
| 54 | RTL8C |  |  | 8375 | -0.380 | -0.6476 | Yes |
| 55 | SH3BGRL |  |  | 8409 | -0.390 | -0.6424 | Yes |
| 56 | FEZ1 |  |  | 8457 | -0.412 | -0.6383 | Yes |
| 57 | SCP2 |  |  | 8513 | -0.442 | -0.6343 | Yes |
| 58 | GNAI2 |  |  | 8540 | -0.452 | -0.6269 | Yes |
| 59 | CTXN1 |  |  | 8543 | -0.453 | -0.6168 | Yes |
| 60 | PFN2 |  |  | 8545 | -0.455 | -0.6065 | Yes |
| 61 | LGALS3 |  |  | 8550 | -0.460 | -0.5964 | Yes |
| 62 | SMDT1 |  |  | 8585 | -0.478 | -0.5893 | Yes |
| 63 | ZNF428 |  |  | 8602 | -0.493 | -0.5798 | Yes |
| 64 | FKBP8 |  |  | 8617 | -0.504 | -0.5698 | Yes |
| 65 | METRN |  |  | 8638 | -0.518 | -0.5602 | Yes |
| 66 | GABARAPL2 |  |  | 8658 | -0.531 | -0.5502 | Yes |
| 67 | LAMTOR4 |  |  | 8660 | -0.532 | -0.5381 | Yes |
| 68 | MARCKS |  |  | 8667 | -0.537 | -0.5265 | Yes |
| 69 | ID3 |  |  | 8676 | -0.544 | -0.5150 | Yes |
| 70 | C1orf122 |  |  | 8686 | -0.548 | -0.5034 | Yes |
| 71 | SRP14 |  |  | 8701 | -0.565 | -0.4921 | Yes |
| 72 | C4orf3 |  |  | 8705 | -0.565 | -0.4794 | Yes |
| 73 | PFDN5 |  |  | 8738 | -0.596 | -0.4694 | Yes |
| 74 | SKP1 |  |  | 8756 | -0.617 | -0.4572 | Yes |
| 75 | BEX3 |  |  | 8759 | -0.619 | -0.4432 | Yes |
| 76 | ATP6V1G1 |  |  | 8766 | -0.625 | -0.4296 | Yes |
| 77 | COMMD6 |  |  | 8772 | -0.628 | -0.4158 | Yes |
| 78 | BRI3 |  |  | 8800 | -0.651 | -0.4039 | Yes |
| 79 | FABP7 |  |  | 8811 | -0.661 | -0.3899 | Yes |
| 80 | FABP5 |  |  | 8829 | -0.684 | -0.3761 | Yes |
| 81 | RPS11 |  |  | 8830 | -0.687 | -0.3604 | Yes |
| 82 | RAB31 |  |  | 8831 | -0.687 | -0.3447 | Yes |
| 83 | BEX1 |  |  | 8833 | -0.689 | -0.3290 | Yes |
| 84 | MARCKSL1 |  |  | 8857 | -0.716 | -0.3152 | Yes |
| 85 | TOMM7 |  |  | 8861 | -0.724 | -0.2990 | Yes |
| 86 | RPS27 |  |  | 8865 | -0.727 | -0.2827 | Yes |
| 87 | PHPT1 |  |  | 8871 | -0.736 | -0.2664 | Yes |
| 88 | C9orf16 |  |  | 8876 | -0.744 | -0.2498 | Yes |
| 89 | RPL34 |  |  | 8877 | -0.744 | -0.2327 | Yes |
| 90 | SNX3 |  |  | 8881 | -0.748 | -0.2160 | Yes |
| 91 | TUBA1A |  |  | 8883 | -0.751 | -0.1989 | Yes |
| 92 | CCNI |  |  | 8902 | -0.773 | -0.1832 | Yes |
| 93 | S100B |  |  | 8908 | -0.784 | -0.1658 | Yes |
| 94 | SERF2 |  |  | 8920 | -0.816 | -0.1484 | Yes |
| 95 | PTMS |  |  | 8921 | -0.820 | -0.1296 | Yes |
| 96 | ATP6V1F |  |  | 8924 | -0.828 | -0.1108 | Yes |
| 97 | OST4 |  |  | 8925 | -0.829 | -0.0919 | Yes |
| 98 | EEF1A1 |  |  | 8958 | -0.915 | -0.0745 | Yes |
| 99 | TPT1 |  |  | 8967 | -0.951 | -0.0537 | Yes |
| 100 | FTH1 |  |  | 8981 | -1.131 | -0.0292 | Yes |
| 101 | FTL |  |  | 8984 | -1.286 | 0.0000 | Yes |
Table: GSEA details [plain text format]

  

Fig 2: LARSSON\_STATE\_6: Random ES distribution      
 Gene set null distribution of ES for **LARSSON\_STATE\_6**
